# Development and Psychometric Testing of the Nurses’ Professional Dignity Scale

**DOI:** 10.3390/nursrep15040127

**Published:** 2025-04-11

**Authors:** Michela Piredda, Maddalena De Maria, Rosario Caruso, Anna Marchetti, Giorgia Petrucci, Anna Cerra, Joyce J. Fitzpatrick, Alessandro Stievano

**Affiliations:** 1 Research Unit of Nursing Science, Department of Medicine and Surgery, Campus Bio-Medico di Roma University, 00128 Rome, Italy; m.piredda@unicampus.it; 2 Department of Life Science, Health, and Health Professions, Link Campus University, 00165 Rome, Italy; m.demaria@unilink.it; 3 Department of Biomedical Sciences for Health, University of Milan, 20133 Milan, Italy; rosario.caruso@unimi.it; 4 Research Unit of Nursing Palliative Care, Fondazione Policlinico Universitario Campus Bio-Medico, 00128 Rome, Italy; a.marchetti@policlinicocampus.it; 5 Research Unit of Orthopaedic and Trauma Surgery, Fondazione Policlinico Universitario Campus Bio-Medico, Via Alvaro del Portillo, 200, 00128 Roma, Italy; g.petrucci@policlinicocampus.it; 6 Patient Care Services, Greenwich Hospital, Yale New Haven Health, Greenwich, CT 06831, USA; anna.cerra@greenwichhospital.org; 7 Frances Payne Bolton School of Nursing, Case Western Reserve University, Cleveland, OH 44106, USA; jjf4@case.edu; 8 Department of Clinical and Experimental Medicine, University of Messina, 98122 Messina, Italy

**Keywords:** nurses, dignity, respect, questionnaires and surveys, statistical factor analysis

## Abstract

**Background/Objectives**: Human dignity is an inalienable value central to human rights and ethics. Professional dignity is pivotal to fostering self-esteem, job satisfaction, and high-quality care in nursing. Despite its importance, no validated tool currently exists to measure nurses’ professional dignity in English-speaking contexts. This study aimed to develop and psychometrically test the Nurses’ Professional Dignity Scale (NPDS). **Methods**: The tool’s development was guided by a theoretical model from a meta-synthesis. A consensus meeting with five nurse researchers identified three core dimensions for the NPDS: Respect, Professional Value, and Appreciation. Nineteen items were initially generated and refined through face and content validity assessments (all item-level content validity indices [I-CVIs] ≥ 0.80; scale-level content validity index/Ave [S-CVI/Ave] = 0.92). Psychometric testing was conducted with 227 nurses across clinical settings in the United States using confirmatory factor analysis (CFA) to validate a three-factor model. **Results**: The CFA confirmed the three-factor model with acceptable fit indices (CFI = 0.938, TLI = 0.923, RMSEA = 0.069), resulting in the retention of 15 items. The scale demonstrated excellent reliability, with composite reliability coefficients of 0.92 for Respect, 0.82 for Professional Value, 0.93 for Appreciation, and 0.91 for the overall scale. **Conclusions**: The NPDS is a valid and reliable measure of nurses’ professional dignity, aligning with theoretical frameworks. It captures both status-dignity and condition-dignity aspects, encompassing respect, professional competence, and societal appreciation, offering a multidimensional structure for assessing individual domains and overall scores. The NPDS contributes to advancing nursing research and practice by addressing workplace dignity, enhancing job satisfaction, and fostering supportive organizational environments that recognize nurses’ professional worth. Future studies are recommended to validate the scale in diverse populations and explore its stability over time through longitudinal research. This study highlights the importance of preserving nurses’ dignity in improving professional identity, workplace environments, and patient care outcomes.

## 1. Introduction

Human dignity represents the essential and inalienable value inherent in every person by virtue of their humanity [1]. The roots of this concept can be traced to the Judeo-Christian belief in the creation of humanity in the image of God, which attributes a divine resemblance to all individuals [2]. Dignity is often interpreted through Kantian ethics, where it is described as “intrinsic, unconditional, and incomparable” [3]. Kant argued that humans are invaluable, irreplaceable, and inherently possess the right never to be treated merely as a means but always as ends in themselves [4]. Consequently, respecting human dignity is a fundamental principle governing human interaction and serves as the cornerstone of human rights [5].

Despite its foundational nature, the concept of dignity remains challenging for many scholars [6,7]. They argue that it lacks a precise definition and fails to provide a robust framework for human rights, potentially undermining their effectiveness [6]. A conceptual framework for dignity has been proposed, encompassing the core concept of status dignity along with condition dignity. Status dignity refers to the ethical principles requiring individuals to receive the obligatory treatment they are entitled to under human rights principles. In contrast, condition dignity pertains to the contexts in which individuals experience the treatment they rightfully deserve [8]. 

Within condition dignity research, several scholars highlight social dignity, which is tied to the appreciation and recognition one earns through interpersonal interactions [9,10]. This perspective identifies social dignity as a pivotal aspect of workplace settings [11]. In nursing, professional dignity is a critical dimension of condition dignity. As Combrinck et al. emphasize, professional dignity is rooted in the perception of one’s worth and recognition from others, including patients, colleagues, and management [7]. When nurses experience disrespect, lack of appreciation, or workplace humiliation, their sense of dignity can be severely compromised, leading to emotional distress and reduced job satisfaction [12]. Conversely, environments that support professional dignity by fostering respect, recognition, and ethical professional autonomy enhance nurses’ confidence and their ability to provide high-quality, compassionate care. These findings highlight the need to move beyond abstract conceptualizations of dignity and toward a more pragmatic framework that acknowledges the social and professional dimensions shaping dignity in nursing practice.

The significance of dignity in nursing is underscored by its prominent position in numerous nursing codes of conduct worldwide. The International Council of Nurses’ code emphasizes the importance of respecting human rights in nursing practice [13]. These rights include the right to life, autonomy, cultural rights, and, importantly, dignity and respectful treatment. 

The concept of professional dignity within nursing has garnered substantial attention and has been a focal point of qualitative exploration [7,10,12,14,15]. According to the meta-synthesis by Sabatino et al. [16], nurses’ professional dignity is a multidimensional concept. While some traits are inherent to human nature—such as the right to be respected and appreciated as a unique individual [17]—others stem from personal values, ethics, and professional standards, including dedication to caregiving and adherence to nursing codes. Moreover, certain qualities may be influenced by the surrounding environment and organizational culture. 

A notable example of such influence is the work and cultural context, which shapes the perception and valorization of body care practices. The nursing profession has historically been undervalued and often associated with routine or low-prestige tasks, despite its crucial role in patient care [18]. This perception is shaped by organizational dynamics, such as hierarchical healthcare structures, limited autonomy for nurses, and broader social and cultural norms that have traditionally framed nursing as a subordinate profession. These factors influence not only how nurses perceive their own professional dignity but also how it is acknowledged by colleagues, patients, and the healthcare system at large [19].

Research suggests that when an individual’s dignity is not upheld, it could lead to emotions such as insecurity, guilt, worthlessness, despair, reduced self-confidence, a desire to leave the profession, and a decline in the quality of patient care [20,21,22]. 

In contrast, nurses’ dignity has been positively associated with self-esteem, job satisfaction, inner spiritual commitment, and self-reported health status [23]. To promote nurses’ professional dignity, it is essential to identify both the factors that hinder it and those that contribute to fostering a healthy work environment. Measuring nurses’ professional dignity is also crucial due to its impact on the quality of patient care [20].

Thus far, two measures of workplace dignity have been developed to assess dignity within work environments, primarily targeting office workers to enhance their quality of work life [19]. In nursing, existing tools focus almost exclusively on how nurses perceive and uphold patient dignity, with little attention given to their own professional dignity [24,25,26]. However, growing evidence suggests that dignity violations in the workplace significantly impact nurses’ well-being, job satisfaction, and quality of care. Therefore, understanding and safeguarding nurses’ professional dignity is pivotal for improving workplace conditions, ensuring high-quality patient care, and fostering a sustainable nursing workforce. This critical gap underscores the urgent need for a dedicated tool designed to accurately assess and address professional dignity in nursing [21,23,27,28]. Therefore, this study aimed to develop the Nurses’ Professional Dignity Scale (NPDS) and test its psychometric properties in an English-speaking context.

## 2. Materials and Methods

### 2.1. Design

According to this study aim, the design included the following two main phases: (1) tool development and (2) psychometric testing of the Nurses’ Professional Dignity Scale. This study was reported following the recommendations for instrument and scale development and testing in accordance with Enhancing the Quality and Transparency Of Health Research (EQUATOR) guidelines [29].

### 2.2. Phase One

Phase one was characterized by four steps. During the first step (conceptualization), a literature review was conducted to identify the main indicators characterizing nursing professional dignity. The second step was a consensus meeting among researchers to discuss and endorse the literature review findings, and the third step was item generation.

#### 2.2.1. Conceptualization 

The development of the NPDS was based on the theoretical model, including seven attributes, which was created in the meta-synthesis of nursing professional dignity by Sabatino et al. [16] and on the related nursing literature.

#### 2.2.2. Consensus Meeting

The researchers met to discuss the literature review findings and assess their suitability to characterize the main characteristics of nurses’ professional dignity in clinical practice. The theoretical model found during the conceptualization phase was shared and discussed within an in-person consensus meeting held among five expert nurse researchers. At the end of the consensus discussion, each expert rated the themes using a four-point Likert scale (from 1 = completely disagree to 4 = completely agree). From the seven attributes of the original theoretical model, three themes were retained for NPDS. The interrater agreement among experts for the themes was computed through Fleiss’ K [30]. The level of agreement between experts in defining each of the three themes as a suitable domain for NPDS was high (‘Self-respect’ domain, Fleiss’ K = 0.92; ‘Respectful interpersonal relationship’ domain, Fleiss’ K = 1.00; and ‘Work environment’ domain, Fleiss’ K = 1.00).

#### 2.2.3. Item Generation

Two authors developed the initial pool of items (n = 19) to operationalize the three domains identified in the consensus meeting, considering the literature review findings. The items were statements drafted to measure specific characteristics of nurses’ professional dignity. The respondent should rate each statement referring to the last three months by using a five-point Likert scale from 1 = Not at all true to 5 = Completely true. Further information, including the full list of the initial 19 items, is available in Table S1, Supplementary File S1.

### 2.3. Phase Two: Psychometric Testing

This phase was conducted from January to August 2021. It included a methodological step for assessing the qualitative and quantitative content validity and one cross-sectional data collection to examine the dimensionality of the newly developed tool. Internal consistency and concurrent validity were also assessed.

#### 2.3.1. Content and Face Validity 

Each item was assessed for content validity in terms of relevance, readability, and comprehensiveness with the Content Validity Index (CVI) by a panel of 10 experts selected through a purposive sampling strategy [31]. The selection of 10 experts was based on recommendations from prior methodological studies that suggest a minimum panel size of 6–10 for reliable content validity assessment [31]. Panelists were required to have at least five years of professional experience in nursing or a related healthcare field and prior experience in evaluating or developing research instruments. 

The experts were identified through professional networks, institutional affiliations, and prior research collaborations to ensure a diverse representation of expertise. All panelists were nurses with at least a master’s degree and included a mix of clinical nurses (n = 4), nurse educators (n = 3), and researchers (n = 3) to ensure a comprehensive perspective. They demonstrated knowledge and understanding of the construct under evaluation of nurses’ professional dignity through their professional qualifications, research expertise, and prior experience in the field. This included familiarity with the relevant literature, practical applications, and theoretical foundations of the construct. Further information is available in Table S2, Supplementary File S1.

The panelists rated the relevance of every item on a 4-point scale, where one represented not relevant, two somewhat relevant, three quite relevant, and four highly relevant. The Item-Level CVI (I-CVI) was calculated for each item as the proportion of panelists rating the item as either 3 or 4. An I-CVI value of 0.78 or higher was considered indicative of good content validity [31]. The Scale-Level CVI (S-CVI) was computed using the averaging method (S-CVI/Ave), which involved calculating the mean of the I-CVI values across all items. Unanimous ratings were additionally computed, i.e., S-CVI (UA). An S-CVI/Ave of 0.90 or higher was deemed acceptable, reflecting overall strong agreement among the panelists. The modified kappa statistic (k*) was computed for each item to adjust for chance agreement. This statistic considers the probability of chance agreement and provides a more rigorous assessment of agreement reliability. A k* value of 0.74 or higher was interpreted as excellent, between 0.60 and 0.74 as good, and between 0.40 and 0.59 as fair. Additionally, face validity was assessed using an open-ended question at the end of the CVI evaluation process: “Are the items clear, relevant, and representative of the construct?”. All 19 items were retained in this phase, as the content validity was achieved (see Tables S1 and S3, Supplementary File S1). Hence, the new 19-item NPDS included three domains: Respect (n = 9 items), Professional value (n = 4 items), and Appreciation (n = 6 items). 

#### 2.3.2. Participants and Research Context

A convenience sample of nurses working in various clinical settings on the East Coast of the United States, including medical/surgical units, oncology, critical care, operating rooms, and pediatrics, was recruited. These settings were part of a hospital recognized for its excellence in nursing practice and patient care. As a Magnet-recognized healthcare facility, this institution demonstrated high standards in professional nursing practice, fostering a collaborative, interdisciplinary approach to care. It is renowned for its emphasis on evidence-based practice, continuous professional development, and innovative patient care solutions. The hospital’s commitment to excellence is reflected in its supportive environment, where nurses are empowered to achieve optimal outcomes for patients across diverse specialties. This setting provided an ideal context for studying nurses’ professional dignity, as it embodies the organizational principles that promote respect, appreciation, and professional value among healthcare professionals. A sample size of at least 200 participants was sought according to the widely accepted rule of thumb of 10 subjects per item and to the minimum number (N = 200) recommended to test dimensionality [32].

#### 2.3.3. Statistical Analysis 

Descriptive statistics were used to describe the sociodemographic characteristics of the participants. Skewness and kurtosis were used to evaluate the normality of items [33]. Since the instrument was theory-based, we examined the dimensionality of the NPDS using a confirmatory approach. Specifically, a confirmatory factorial analysis (CFA) was conducted using the maximum likelihood robust estimator to account for the non-normal distribution of the items [34]. To evaluate the adequacy of the tested model, a multifaceted approach [35] was used considering the following goodness-of-fit indices: confirmatory fit index (CFI) [36] and the Tucker–Lewis index (TLI) [37], in which the values of 0.90–0.95 indicate acceptable fit, and values > 0.95 indicate excellent fit; standardized root mean square residual (SRMR), in which the values of equal to or less than 0.08 indicate a good fit; and root mean square error of approximation (RMSEA), in which the values of less than 0.06 indicate a good fit. Chi-square tests were interpreted together with the above indices. Factor loadings of >0.30 were considered adequate [38,39]. The reliability was estimated with composite reliability coefficients [40], factor score determinacy [34], and Bentler’s model-based internal consistency reliability considering the multidimensionality of the scale [41]. Values ≥ 0.70 were considered adequate [42]. Item discrimination was estimated with a corrected item-total correlation coefficient [43], considering values ≥ 0.20 as adequate [44]. The level of significance was set at ≤0.05. Statistical analyses were performed using Mplus Version 8.4 [34].

### 2.4. Ethical Considerations 

This study was conducted in accordance with the Declaration of Helsinki [45] and approved by the Institutional Review Board of the Centre of Excellence for Nursing Scholarship of OPI Rome Protocol number 3.21.2. Potential participants were approached by researchers and provided comprehensive written and verbal information about the study methods and aims before taking part in this study. They were assured that they could withdraw from this study at any time or even ask that the questionnaire not be used in the research. Additionally, they were assured that the data collected from participants (i.e., gender and age) would be treated confidentially and would be analyzed and reported in an aggregated way. Informed written consent was granted for study participation and data handling by all participants before completing and delivering the questionnaire. Personal data collected

## 3. Results

### 3.1. Content Validity

The results of the content validity analysis, including individual item-level CVI values, modified kappa statistics, and scale-level CVI metrics, are detailed in Tables S1 and S3, Supplementary File S1. 

### 3.2. Sample

As shown in Table 1, the sample included 227 nurses, mostly female (97.4%), with a Bachelor of Nursing Science (85.7%), Associate degree (10.3%), or Diploma (3.6%). A master’s degree was held by 35 nurses (15.6%), and a Doctor of Nursing Practice (DNP) by 4 participants (1.8%). The average work experience was 20.31 years. Nurses were working on the East Coast of the United States in clinical settings such as medical and surgical wards, oncology, operating rooms, telemetry, transplant, IV teams, critical care areas (e.g., ICU, NICU), antepartum maternity, labor and delivery, and pediatrics.

### 3.3. Item Description 

The items with the highest scores were Item #4, “I consider individual respect very important” (mean 4.69, SD 0.54), and #1, “I always respect myself as a person” (mean 4.43, SD 0.73). The items with the lowest scores were Item #9, “Healthcare assistants respect nurses’ knowledge and assignments in relation to patients” (mean 3.86, SD 0.81), and Item #13, “My work as a nurse provides me with financial independence” (mean 3.77, SD 1.05). Several items (#1, #4, #10, #11, #12, and #14) were non-normally distributed, with skewness and kurtosis > |1|. The corrected item total correlations were >0.43. A detailed description of the item is presented in Table 2.

### 3.4. Dimensionality

Since nurses’ professional dignity is described as comprising “respect” (n = 9 items), “professional values” (n = 4 items), and “appreciation” (n = 6 items), a three-factor confirmatory model was specified for the 19-item NPDS. The model yielded an inadequate fit (*χ*^2^ (149, N = 149) = 612.990, *p* < 0.001, CFI = 0.770, TLI = 0.736, RMSEA = 0.117 (90% CI = 0.108 0.127), *p* < 0.001, SRMR = 0.078), and four items were removed as their loadings were <0.30 and not significant.

The three-factor confirmatory model for the resulting 15-item NPDS had a poor fit: *χ*^2^ (87, N = 227) = 267.338, *p* < 0.001, CFI = 0.876, TLI = 0.851, RMSEA = 0.096 (90% CI = 0.083 0.109), *p* < 0.001, SRMR = 0.056. Inspection of the modification indices revealed that the misfit was caused by excessive covariance between items #1 (I always respect myself as a person) and #4 (I consider individual respect very important), #2 (My work colleagues respect me as a person), #5 (All other healthcare professionals value me as a nurse), #3 (Patients respect me as a person), and #8 (Patients value my work). The excess in the covariance detected by the covariance of the error of items #1 and #4 and #3 and #8 could be attributed to the respect for one’s work as a source of appreciation, respect, and trust addressed by all these items. The surplus error covariance between items #2 and #5 may be due to phrasal semantics as follows: respect and value address a positive consideration of the individual as both a person and a professional. Thus, when we reran the model, the residuals of these items were correlated [46,47]. The model fit was good: *χ*^2^ (84, N = 227) = 173.943, *p* < 0.001, CFI = 0.938, TLI = 0.923, RMSEA = 0.069 (90% CI = 0.054 0.083), *p* = 0.018, SRMR = 0.046.

**Figure 1:**
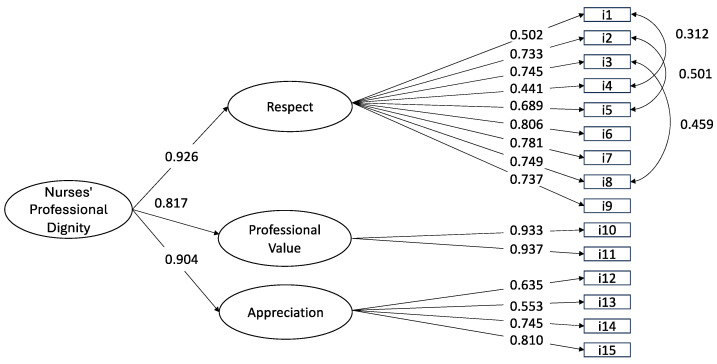
Factorial structure of the 15-item Nurses’ Professional Dignity Scale at confirmatory factor analysis (n = 227 nurses).

Since the three factors were significantly correlated (ranging from 0.739 to 0.838, *p* < 0.001), a second-order hierarchical model was examined, and the same fit as the first-order one was produced. This final model showed that although the NPDS scale was multidimensional at the level of primary factors, it was unidimensional at the level of second-order factors. All factor loadings were significant and higher than 0.5 (see Figure 1).

### 3.5. Reliability

Composite reliability was 0.92 for the Respect, 0.82 for the Professional value, 0.93 for the Appreciation factor, and 0.91 for the entire scale, showing excellent internal consistency. The factor score determinant coefficient was 0.97 for the Respect, 0.89 for the Professional value, 0.97 for the Appreciation factor, and 0.89 for the entire scale. The internal consistency for the second-order factor structure, which was estimated with Bentler’s model-based internal consistency, showed a high coefficient of 0.91 in the NDPS. These results support the use of scores for each factor and the combined scores of the 15 items of the NDPS. See Table 3 for detailed reliability data regarding individual NPDS factors and the overall scale. 

## 4. Discussion

This study aimed to develop and psychometrically test the NPDS for English-speaking contexts. The structural validity of the NPDS was confirmed as a three-factor model, consistent with the theoretical framework on which it was based [16]. The CFA demonstrated good fit indices and high, significant loadings. Their semantic and conceptual similarities justify the covariance specified between several items. Consequently, the NPDS is a valid and reliable instrument for measuring the professional dignity of English-speaking nurses. While the NPDS is multidimensional at the level of first-order factors, it is unidimensional at the level of the second-order factor, allowing for the computation of an overall scale score in addition to scores for the three individual factors. The final 15-item NPDS, along with response options and scoring information, is provided within Table 2.

The three factors—respect, professional value, and appreciation [16]—align with the classification of professional dignity as status-dignity (professional value) and condition-dignity (respect and appreciation) within the conceptual framework for dignity proposed by Gilabert [8]. Respect pertains to nurses’ perception of being respected by others, which contributes to professional dignity and competence. This is consistent with findings by Stievano et al. [10], who highlighted that respectful communication between nurses and other healthcare professionals enhanced nurses’ sense of dignity and fostered positive and respectful relationships with patients. 

Professional dignity encompasses respectful interactions, participation, equality, intrinsic value, and public appreciation [48]. Workplace dignity is self-recognized and externally acknowledged, as it depends on individuals’ perception of their worth and how others recognize it through respect and trust in interactions [48]. Conversely, a lack of respect in communication—gossip, the sharing of false information, sarcastic comments, and critical attitudes—and a lack of recognition for one’s contribution are among the most common negative interactions experienced by nurses. Such behaviors can cause significant stress and frustration, hindering the development and preservation of professional dignity [27,49].

The professional value of nurses was operationalized using two indicators: the meaning attached to their work and the perceived ability of their work as nurses to make a meaningful difference within the healthcare organization. Nurses’ professional value is closely associated with the dignity and respect they earn when practicing with professional knowledge and competence. This reflects the core values of professional identity and elevates the dignity of the nursing profession [14]. 

In recent decades, however, the social stigmatization of care-dependent patients has extended to those who provide their care, devaluing and rendering the work of nurses invisible [50,51]. Similarly, prejudices surrounding body care have further hindered the social value of the nursing profession. Work involving body care has historically been marginalized, often perceived as “dirty” or undignified, and surrounded by silence. This perception relegates body care to a low-prestige domain despite its central role in ensuring the dignity and well-being of individuals. These societal attitudes have limited an unbiased exploration of the body’s profound meaning and intrinsic value as an object of care and a symbol of humanity [19,52]. Therefore, a serious and comprehensive reflection on the importance of the nursing profession is necessary, recognizing the dignity of the bodies being cared for and those who provide such care with competence and sensitivity [19]. 

The impact of a positive and supportive practice environment on nurses’ professional dignity has been well-documented [27]. Research highlights the association between recognizing nurses’ professional dignity and competence and fostering a positive healthcare organizational climate that enhances job satisfaction and professional motivation [21,27]. In contrast, less healthy organizational environments, where respect for professional dignity is absent, lead to decreased job satisfaction and increased stress levels [53,54]. For instance, imbalanced nurse-to-patient ratios and heavy workloads present significant challenges, hindering nurses’ ability to deliver the high-quality care their roles demand [55].

Appreciation refers to nurses’ perception of the respect they hold for themselves and the recognition they receive from society, relatives, and friends. The social acknowledgement of nurses’ contributions is critical to their professional worth and self-esteem [21,56]. When recognition and appreciation are lacking, a foundational element of nurses’ personal and professional development is missing [57], which can result in diminished professional value, interest, and motivation [58].

A tool specifically designed to measure nurses’ professional dignity, the Perceived Clinical Nurses’ Professional Dignity Scale (PCNPDS), was recently developed in Iran and tested with 500 hospital nurses [56]. However, the article describing this tool was published after our study, so we were unaware of its existence during the development of our own tool. The 22-item PCNPDS was developed using a hybrid concept analysis, incorporating theoretical research, fieldwork, and final analysis. It was validated through exploratory (N = 300) and confirmatory factor analysis (N = 200), identifying a three-factor model: organizational dignity (10 items), dignity-based competency (8 items), and dignity-based appreciation (4 items). Its reliability, assessed through Cronbach’s alpha, McDonald’s omega, and intraclass correlation coefficients, was excellent (0.901, 0.898, and 0.96, respectively). 

When comparing the NPDS and the PCNPDS, many similarities emerge regarding factors and their constituent items. The NPDS factor ‘appreciation’ closely aligns with ‘dignity-based appreciation’ in the PCNPDS. In contrast, the NPDS factor ‘respect’ is reflected in the PCNPDS factor ‘dignity-based competency’, which includes three items related to respect. The NPDS factor ‘professional value’ is comparable to the PCNPDS factor ‘organizational dignity’. However, the PCNPDS includes a significantly larger number of items within this factor (10 versus 2), suggesting stronger cultural differences between the 2 countries. Overall, the similarities between the two tools provide further support for their validity in measuring nurses’ professional dignity across different cultural contexts. The NPDS offers a concise yet robust measure of the overall construct of nurses’ professional dignity and its individual dimensions—respect, professional value, and appreciation—with only 15 items.

### 4.1. Strengths and Limitations

The strengths of this study lie in the rigorous methodological steps followed for the instrument’s development and the robust statistical analyses performed. The development of NPDS was grounded in a comprehensive review of relevant expert consensus, ensuring the inclusion of the most pertinent aspects of professional dignity in nursing. However, this study has limitations that must be considered when interpreting the results. This study was conducted with a convenience monocentric sample, which limits the generalizability of the findings to the broader nursing population. Future research involving more diverse and representative samples is necessary to validate the scale’s applicability across various settings and contexts. Moreover, a limitation of the instrument lies in its self-report nature, as it considers only nurses’ perception of professional dignity. Future studies should incorporate tools that gather objective data on nurses’ professional dignity from organizational environments and from other healthcare professionals and patients.

### 4.2. Implications for Practice and Research

The development and validation of the NPDS hold significant implications for nursing practice and research. In clinical practice, the NPDS provides a concise and reliable tool for assessing professional dignity, enabling healthcare organizations to identify areas requiring improvement in workplace dynamics, nurse-patient interactions, and organizational culture. Healthcare administrators could be able to foster supportive environments that promote job satisfaction, motivation, and the overall quality of care delivery by addressing the dimensions of respect, professional value, and appreciation.

From a research perspective, the NPDS creates opportunities to explore the relationships between professional dignity and other critical variables, such as burnout, retention rates, and organizational outcomes. Additionally, it facilitates cross-cultural comparisons, enabling researchers to examine how cultural nuances influence nurses’ perceptions of dignity. Future studies should focus on applying the NPDS in diverse settings and populations to establish broader validity and reliability.

Evaluating the stability of the instrument over time through test-retest studies and conducting longitudinal research could provide valuable insights into the impact of interventions aimed at improving respect, appreciation, and professional value on nurse well-being and patient outcomes. International studies are needed to enable cross-cultural validation of the new instrument, contributing to its refinement and adaptation for global contexts. Furthermore, comparing the NPDS with tools such as the PCNPDS may offer a deeper understanding of cultural dimensions of dignity in nursing, further informing the development of interventions and measures.

## 5. Conclusions

This study successfully developed and psychometrically tested the NPDS for English-speaking nurses. The NPDS demonstrated excellent structural validity and reliability, confirming its utility as a multidimensional measure of professional dignity encompassing the dimensions of respect, professional value, and appreciation. These findings align with existing frameworks on workplace dignity and underscore the multidimensional nature of professional dignity in nursing.

The NPDS offers healthcare organizations a practical and concise tool to evaluate and enhance nurses’ experiences of dignity within their professional roles. While some cultural differences were noted between the NPDS and the PCNPDS, the similarities in factor structures highlight the universal importance of dignity in nursing. Nevertheless, the study’s findings must be interpreted cautiously due to the monocentric sample, necessitating further validation in broader, more diverse populations. The NPDS represents a significant contribution to nursing by addressing the critical need for a reliable and valid measure of professional dignity. This tool supports theoretical exploration and practical interventions to foster dignity in nursing environments and enhance outcomes for nurses and patients alike. 

## Figures and Tables

**Table 1:** Participants sociodemographic characteristics (N = 227 nurses).

Variable	N (%)
Gender	
Female	221 (97.4%)
Male	6 (2.6%)
Education	
Bachelor of Science in Nursing	192 (85.7%)
Associate degree	23 (10.3%)
Diploma	8 (3.6%)
No answer	4 (0.4%)
Master’s Degree	35 (15.4%)
Doctorate of Nursing Practice (DNP)	4 (1.8%)
Work experience	20.31 years

**Table 2:** NPDS item descriptive characteristics (N = 227 nurses).

Items	Mean	SD	Skewness	Kurtosis	ITC
Q1 I always respect myself as a person	4.43	0.725	−1.137	0.872	0.482
Q2 My work colleagues respect me as a person	4.22	0.761	−0.875	0.69	0.695
Q3 Patients respect me as a person	3.99	0.884	−0.761	0.39	0.740
Q4 I consider individual respect very important	4.69	0.535	−1.881	4.649	0.433
Q5 My nurse colleagues value me as a nurse	4.31	0.677	−0.821	0.928	0.663
Q6 All other healthcare professionals value me as a nurse	3.97	0.831	−0.656	0.555	0.776
Q7 Nursing management (head nurses, managers, etc.) value me as a nurse	4.09	0.881	−1.018	0.965	0.729
Q8 Patients value my work	4.06	0.816	−0.662	0.295	0.737
Q9 Healthcare assistants respect nurses’ knowledge and assignments in relation to patients	3.86	0.807	−0.433	−0.159	0.684
Q10 My work as a nurse has meaning for my organization (e.g., healthcare facility)	4.07	0.943	−1.196	1.557	0.703
Q11 My work as a nurse makes a difference within my organization (e.g., healthcare facility)	4.10	0.937	−1.319	1.934	0.715
Q12 My work as a nurse increases my sense of respect for myself	4.29	0.808	−1.296	2.144	0.562
Q13 My work as a nurse provides me with financial independence	3.77	1.053	−0.555	−0.515	0.471
Q14 My family, relatives, and friends appreciate the value of my work	4.44	0.758	−1.623	3.54	0.635
Q15 Society appreciates the value of my work	4.03	0.911	−0.875	0.616	0.690

Legend. ITC: corrected item total correlation; SD: standard deviation; Q1-Q15: NPDS items.

**Table 3:** Internal consistency reliability of NPDS factors and overall scale.

Factors	Composite Reliability	Factors Score Determinacies	Bentler’s Model-Based Internal Consistency
F1 Respect	0.92	0.97	
F2 Professional Value	0.82	0.89	
F3 Appreciation	0.93	0.97	
NPDS	0.91	0.89	0.91

## Data Availability

The data that support the findings of this study are available on request from the corresponding author.

## Supplementary Materials

The following supporting information can be downloaded at: https://www.mdpi.com/article/10.3390/nursrep15040127/s1, Supplementary File S1: Content validity: panelists and ratings. Table S1. Content validity indices; Table S2. Characteristics of the 10 panelists; Table S3. Scale-level content validity index.

## Public Involvement Statement

No public involvement in any aspect of this research.

## Guidelines and Standards Statement

This manuscript adheres to STROBE guidelines for observational studies.

## Abbreviations

The following abbreviations are used in this manuscript:

NPDS	Nurses’ Professional Dignity Scale
I-CVI	Item-Level Content Validity Index
S-CVI	Scale-Level Content Validity Index
S-CVI/Ave	Scale-Level Content Validity Index (Averaging Method)
CFA	Confirmatory Factor Analysis
CFI	Confirmatory Fit Index
TLI	Tucker–Lewis Index
RMSEA	Root Mean Square Error of Approximation
SRMR	Standardized Root Mean Square Residual
CVI	Content Validity Index
k*	Modified Kappa Statistic
ICU	Intensive Care Unit
NICU	Neonatal Intensive Care Unit
DNP	Doctor of Nursing Practice
PCNPDS	Perceived Clinical Nurses’ Professional Dignity Scale
EQUATOR	Enhancing the QUAlity and Transparency Of health Research
UA	Universal Agreement
