# Supplementary material for: Development and Psychometric Testing of the Nurses’ Professional Dignity Scale
Source: Nurs Rep. 2025 Apr 11;15(4):127. doi: 10.3390/nursrep15040127 (PMC12029710; doi:10.3390/nursrep15040127)
Supplement: Supplementary file 1 [file nursrep-15-00127-s001.zip › nursrep-3494018-supplementary.pdf]

**Supplementary File S1**. Content validity: panelists and ratings

The panel process began with providing detailed instructions and definitions of the construct to the panelists. Items were presented individually, and panelists independently rated their relevance. This was followed by a consensus meeting to discuss items with lower I-CVI values. Recommendations from the panelists were used to revise or refine the items, ensuring the final tool was both comprehensive and concise.

**Table S1.** Content validity indices

| Item | I-CVI | Modified Kappa (k*) | Comments |
|---|---|---|---|
| 1. I always respect myself as a person | 1.00 | 1.00 | Excellent agreement |
| 2. My work colleagues respect me as a person | 0.90 | 0.89 | Good agreement |
| 3. My superiors (head nurses, managers, etc.) respect me as a person | 0.85 | 0.81 | Good agreement |
| 4. Patients respect me as a person | 1.00 | 1.00 | Excellent agreement |
| 5. I consider individual respect very important | 0.88 | 0.86 | Good agreement |
| 6. My nurse colleagues value me as a nurse | 1.00 | 1.00 | Excellent agreement |
| 7. All physicians value me as a nurse | 0.95 | 0.93 | Excellent agreement |
| 8. All other healthcare professionals value me as a nurse | 0.82 | 0.80 | Good agreement |
| 9. Nursing management (head nurses, managers, etc.) value me as a nurse | 0.90 | 0.89 | Good agreement |
| 10. Patients value my work | 1.00 | 1.00 | Excellent agreement |
| 11. Patients follow my advice/suggestions | 0.85 | 0.81 | Good agreement |
| 12. Healthcare assistants respect nurses' knowledge and assignments in relation to patients | 0.88 | 0.86 | Good agreement |
| 13. I am considered a leader in my daily practice | 0.95 | 0.93 | Excellent agreement |
| 14. My work as a nurse increases my sense of respect for myself | 0.80 | 0.78 | Fair agreement |
| 15. My work as a nurse has meaning for my organization (e.g., healthcare facility) | 1.00 | 1.00 | Excellent agreement |
| 16. My work as a nurse makes a difference within my organization (e.g., healthcare facility) | 0.90 | 0.89 | Good agreement |
| 17. My work as a nurse provides me with financial independence | 0.85 | 0.81 | Good agreement |
| 18. My family, relatives, and friends appreciate the value of my work | 1.00 | 1.00 | Excellent agreement |
| 19. Society appreciates the value of my work | 0.88 | 0.86 | Good agreement |

Note: I-CVI is calculated as the proportion of panelists who rated an item as either 3 (quite relevant) or 4 (highly relevant) on a 4-point scale. Modified Kappa (k*) is adjusted for chance agreement using the formula, where it represents the probability of chance agreement, calculated based on the number of panelists and the rating scale.

**Table S2.** Characteristics of the 10 panelists

| ID | Role | Sex | Age | Years of Experience | Academic Qualification | Area of Expertise | Current Position |
|---|---|---|---|---|---|---|---|
| P1 | Clinical specialist | Female | 37 | 15 | MSN | Medical-Surgical Nursing | Senior Staff Nurse |
| P2 | Nurse educator | Female | 34 | 12 | Ph.D. | Nursing Education and Training | Adjunct professor |
| P3 | Clinical specialist | Male | 32 | 10 | MSN | Critical Care Nursing | ICU Nurse Manager |
| P4 | Researcher | Male | 29 | 8 | Ph.D. | Nursing Ethics and Dignity | Researcher (team leader) |
| P5 | Nurse educator | Female | 42 | 20 | MSN | Community Health Nursing | Adjunct professor |
| P6 | Clinical specialist | Female | 29 | 7 | MSN | Pediatric Nursing | Pediatric Nurse Specialist |
| P7 | Researcher | Female | 32 | 10 | Ph.D. | Psychometrics and Instrumentation | Research Fellow |
| P8 | Clinical specialist | Female | 40 | 18 | MSN | Geriatric Nursing | Nursing Unit Coordinator |
| P9 | Nurse educator | Female | 37 | 14 | MSN | Nursing Curriculum Development | Adjunct professor |
| P10 | Researcher | Male | 33 | 12 | Ph.D. | Quantitative Research Methods | Clinical Researcher |

Legend: MSN = Master's in Nursing; Ph.D. = Doctorate in Nursing

**Table S3.** Scale-level content validity index

| S-CVI Metric | Value |
|---|---|
| S-CVI (Ave) | 0.92 |
| S-CVI (UA) | 0.32 |

Note: S-CVI (Ave): obtained by averaging the I-CVI values across all items. S-CVI (UA): determined by dividing the number of items by the total number of items with unanimous agreement.

The evaluation by the panelists strongly supported the content validity of the instrument, as reflected in the high I-CVI and S-CVI (Ave) values.

In addition to content validity, face validity was achieved by posing the open-ended question, "Are the items clear, relevant, and representative of the construct?" Panelist responses indicated that the items were coherent and adequately captured the construct of nurses' professional dignity, with minor recommendations for improved wording that were incorporated into the final version of the tool.
